# A novel paradigm for fast training data generation in asynchronous movement-based BCIs

**DOI:** 10.3389/fnhum.2025.1540155

**Published:** 2025-02-11

**Authors:** Markus R. Crell, Kyriaki Kostoglou, Kathrin Sterk, Gernot R. Müller-Putz

**Affiliations:** ^1^Institute of Neural Engineering, Graz University of Technology, Graz, Austria; ^2^BioTechMed Graz, Graz, Austria

**Keywords:** electroencephalography, self-paced brain-computer interface, cue-based paradigm, movement-related cortical potential, asynchronous detection

## Abstract

**Introduction:**

Movement-based brain-computer interfaces (BCIs) utilize brain activity generated during executed or attempted movement to provide control over applications. By relying on natural movement processes, these BCIs offer a more intuitive control compared to other BCI systems. However, non-invasive movement-based BCIs utilizing electroencephalographic (EEG) signals usually require large amounts of training data to achieve suitable accuracy in the detection of movement intent. Additionally, patients with movement impairments require cue-based paradigms to indicate the start of a movement-related task. Such paradigms tend to introduce long delays between trials, thereby extending training times. To address this, we propose a novel experimental paradigm that enables the collection of 300 cued movement trials in 18 min.

**Methods:**

By obtaining measurements from ten participants, we demonstrate that the data produced by this paradigm exhibits characteristics similar to those observed during self-paced movement.

**Results and discussion:**

We also show that classifiers trained on this data can be used to accurately detect executed movements with an average true positive rate of 31.8% at a maximum rate of 1.0 false positives per minute.

## 1 Introduction

For the control of applications with a brain-computer interface (BCI), intuitive control is important to achieve long-term acceptance among users. Particularly individuals with motor disabilities such as locked-in syndrome require an accessible alternative to conventional communication methods. While electroencephalography (EEG) -based BCIs utilizing modalities such as visual evoked potentials (VEPs) or P300 offer high accuracy for the selection of targets (Edelman et al., 2024), these systems rely on externally generated signals and require users to be attentive to the displayed screen. In comparison, the utilization of executed, imagined or attempted movement as a control signal poses lower burden on the users since it resembles natural processes (Müller-Putz et al., 2016; Edelman et al., 2024; Müller-Putz et al., 2021) and does not necessarily require overt attention.

The utilization of neural correlates of movement as a control signal relies on the detection of motor-related dynamics in the neural signals. When multiple movements need to be differentiated, this involves categorization of the detected motion. This detection of movement events, which are not necessarily time-locked to certain external events, is defined as asynchronous movement detection. A limited number of studies have investigated the asynchronous detection of movements from EEG, most of which focus on motor imagery (Müller-Putz et al., 2010; Niazi et al., 2013; Lin et al., 2016; Niazi et al., 2011; Pfurtscheller and Solis-Escalante, 2008; Townsend et al., 2004; Pereira et al., 2018) with some addressing movement execution (Zhang et al., 2024; Niazi et al., 2013; Lin et al., 2016; Niazi et al., 2011; Fatourechi et al., 2007, 2008; Hasan and Gan, 2010; Xu et al., 2014; Liu et al., 2018) and attempts (Ofner et al., 2019).

Most models that are used for asynchronous movement detection need to be trained on labeled data and therefore require knowledge of the exact timing of the movement. While for non-disabled participants executed movements can be tracked with electromyography or motion capture technology and the actual movement onset can be assigned to the neural data, the generation of labeled training data for individuals with impaired movement requires the attempt or imagination of the movement based on a cue of which the exact timing is known. Traditionally, visual cues are elicited on a display and participants execute or attempt/imagine the corresponding movement based on this cue. However, Ofner et al. (2019) showed that the usage of abrupt visual cues elicits neural patterns which alter EEG signals, thereby producing a superposition of visual and motor potentials during the movement which differs from the neural data elicited during self-paced motion. These differences result in reduced performance when training models to detect neural correlates of movement during self-paced action. Ofner et al. (2019) also showed that fading cues diminish these visual effects and should preferably be used when collecting training data for asynchronous movement-based BCIs. Suwandjieff and Müller-Putz (2024) further investigated the neural correlates elicited during cue-based movement tasks and additionally investigated the effects of different cues on the timing of the actual movement onset relative to the intended onset. Especially for motor-impaired patients which cannot execute movements, it is crucial that the actual onset of the movement attempt is accurately aligned with the cue. While a constant delay between cue and movement does not pose any challenges, it is important to minimize the standard deviation of the difference between movement onset and cue in order to extract more consistent and reliable neural patterns. Suwandjieff and Müller-Putz (2024) determined that out of multiple investigated cues, a rotational cue was the most effective in reducing visual cue interference and minimizing variability in movement onset timings relative to the cue. For this cue, a rotating cross was displayed over a fixed cross and participants performed a movement whenever rotating and fixed cross overlapped. Traditional cue-based paradigms for generating training data, such as the one described in Suwandjieff and Müller-Putz (2024) and Ofner et al. (2019), typically lead to longer training periods as the time between the cue symbols being presented and the actual cue for executing the movement must be sufficiently long to allow for VEPs to subside. Durations of more than 8 s are common in these paradigms. This suboptimal characteristic of traditional cue-based paradigms facilitates the adaptation and implementation of new, improved paradigms.

In the current study, we opted to design a paradigm focused on minimizing visual effects while maintaining short inter-trial intervals (ITI). We hypothesized that extending the existent rotational cue by incorporating a continuous rotation at varying rotational speed would minimize visual cue effects since no abrupt visual cues are introduced during the waiting phase for the next motion. Further, since the constant rotation enables the consecutive execution of movements, the paradigm allows for the reduction of ITI compared to traditional cue-based paradigms. We hypothesized that this fast generated training data could be used to train a decoder to detect the executed motions during self-paced movement.

## 2 Materials and methods

### 2.1 Participants and setup

**Figure 1:**
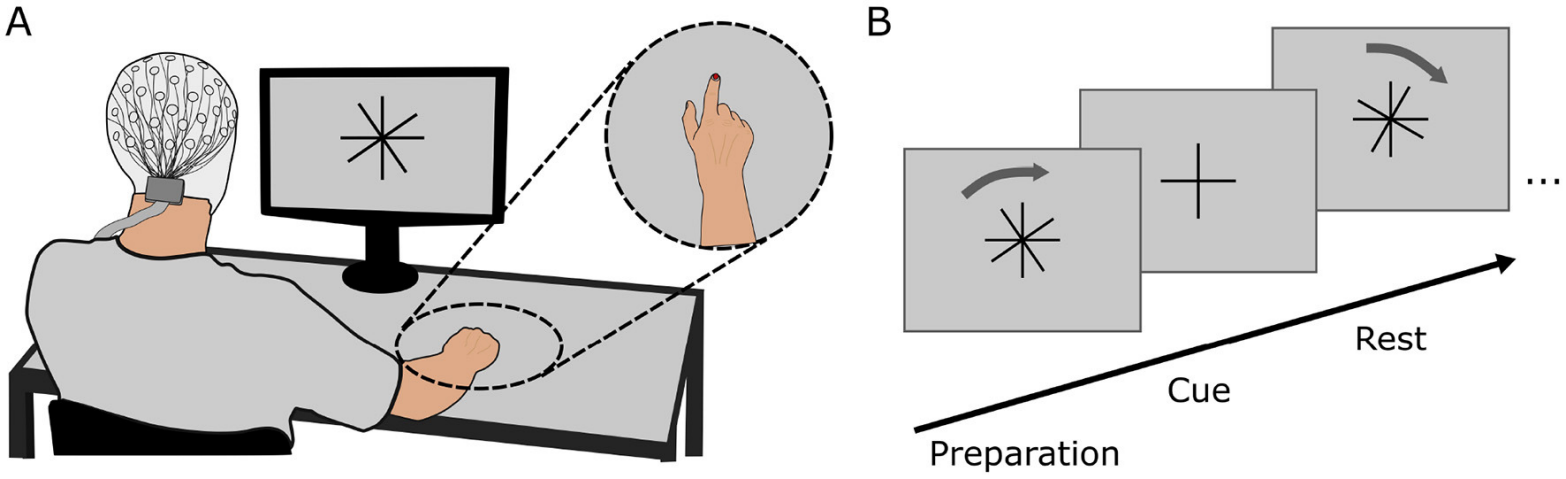
**(A)** Complete setup of the experiment comprising the EEG cap, display and motion capture marker. Participants were seated comfortably at a distance of 80 cm in front of the display on which the paradigm was shown. **(B)** Experimental paradigm for generating cued training data. The overlaid cross (rotation cross) rotated in clockwise direction relative to the fixed cross (fixation cross). Participants were instructed to execute the movement whenever both crosses overlapped.

Ten healthy, right-handed participants (6 female) with normal or corrected-to-normal vision took part in the study. Participants gave written informed consent prior to the study and received monetary compensation for their time. The study was approved by the Ethics Committee at Graz University of Technology and was conducted in accordance with the Declaration of Helsinki. One participant was excluded from further processing due to problems during the recording. Participants were seated comfortably in front of a display (distance: 80 cm) and were equipped with a cap containing 60 EEG electrodes positioned according to the 10 − 10 system (Oostenveld and Praamstra, 2001). Four electrooculography (EOG) electrodes were attached on the outer canthi of the eyes and above and below the left eye. The right hand of the participants was placed on a table in a natural resting position (palm down). A colored marker was attached to the nail of the index finger of the participants to allow for motion tracking of the finger. The complete setup is given in Figure 1A. Participants further had access to a keyboard which they could position in reach of their left hand.

EEG and EOG data was recorded with biosignal amplifiers (BrainAmp, Brain Products GmbH, Gilching, Germany) at a rate of 500 Hz. The position of the index finger was recorded via motion capture algorithms that tracked the marker position and was sampled at a rate of 30 Hz.

### 2.2 Experimental paradigm

Eye-movement data was collected during a specific eye-movement paradigm prior to the main experimental paradigm to enable eye-artifact correction in the neural data. We utilized the eye-movement paradigm from Kobler et al. (2020) in which participants follow a circle on the screen to perform predefined horizontal and vertical eye movements as well as blinks and rest periods with their eyes. The eye-movement paradigm had a duration of approximately 7 min.

After this, the main experimental paradigm was displayed on a screen in front of the participants. The paradigm comprised four runs, of which the first three runs were *cue-based runs* and the fourth run was a *self-paced run*. Between each run the paradigm execution was halted to allow for breaks of variable length. Participants could continue with the paradigm by pressing the space bar as soon as they felt sufficiently prepared. During each cue-based run, two similar crosses were shown on the display in front of the participant: one cross (fixation cross) was fixed to an upright position while the other cross (rotation cross) was overlaid to the fixation cross and rotated at a predefined velocity. At the beginning of every run, the rotation cross was tilted by 10° compared to the fixation cross. After pressing the space bar to continue with or start the paradigm, both crosses were statically displayed for 1.5 s before the rotation cross started to rotate to prepare participants for the upcoming movement. Participants were instructed to execute a flexion movement of all fingers of the right hand including the thumb to form a fist whenever the fixation and rotation crosses overlapped. Instructions were given to start the movement as soon as the crosses overlapped, maintain the flexion for 500 ms and then return the fingers to the original resting position. The angular velocity at which the rotation cross rotated was varied continuously to allow for variable inter-trial intervals.


(1) $$\begin{array}{l} \omega \left(t\right)=\frac{90^{◦}}{T_{ITI}}+A\cdot \sin\left(2\pi \cdot a_{base}\cdot t\right)\cdot \sin\left(2\pi \cdot a_{mod}\cdot t+\varphi \right) \end{array}$$

The velocity was chosen according to Equation 1 where *A* determines the spread of the ITI and *a*_*base*_, *a*_*mod*_ and φ the speed of the change in velocity. The mean ITI is defined by *T*_*ITI*_. This equation ensured that the changes in velocity were continuously differentiable and no abrupt change in the velocity of the rotation occurred. The average inter-trial interval was *T*_*ITI*_ = 3.3 s. Based on visual inspection of the resulting angular velocity and the distribution of the inter-trial intervals, values were selected as *A* = 10.24, *a*_*base*_ = 0.032, *a*_*mod*_ = 0.016 and φ = 28.1 which resulted in a minimum and maximum ITI of 2.50 and 4.75 s. We opted for a slow variation in the inter-trial intervals over the course of one run such that the angular velocity would not change abruptly within one trial while also providing distributed slow and fast trials over the run. Each run contained 100 trials, thus lasting for a duration of 333.9 s when accounting for additional times at the start and end of a run. At the end of a run, the crosses were faded out 10° before the next overlap and a break screen was displayed. The output of the paradigm on the display is shown in Figure 1B.

During the self-paced run, only the fixation cross was displayed. Participants were instructed to execute flexion movements similar to those of the cue-based runs at times of their choosing. They were asked to alternate between periods of fast consecutive movements and periods of long breaks with about 10 s between each motion. Further, participants were instructed to maintain an average ITI consistent with that of the cue-based runs. The duration of the self-paced run was similar to that of the individual cue-based runs. The number of trials in a self-paced run was thus not fixed.

### 2.3 Data processing

**Figure 2:**
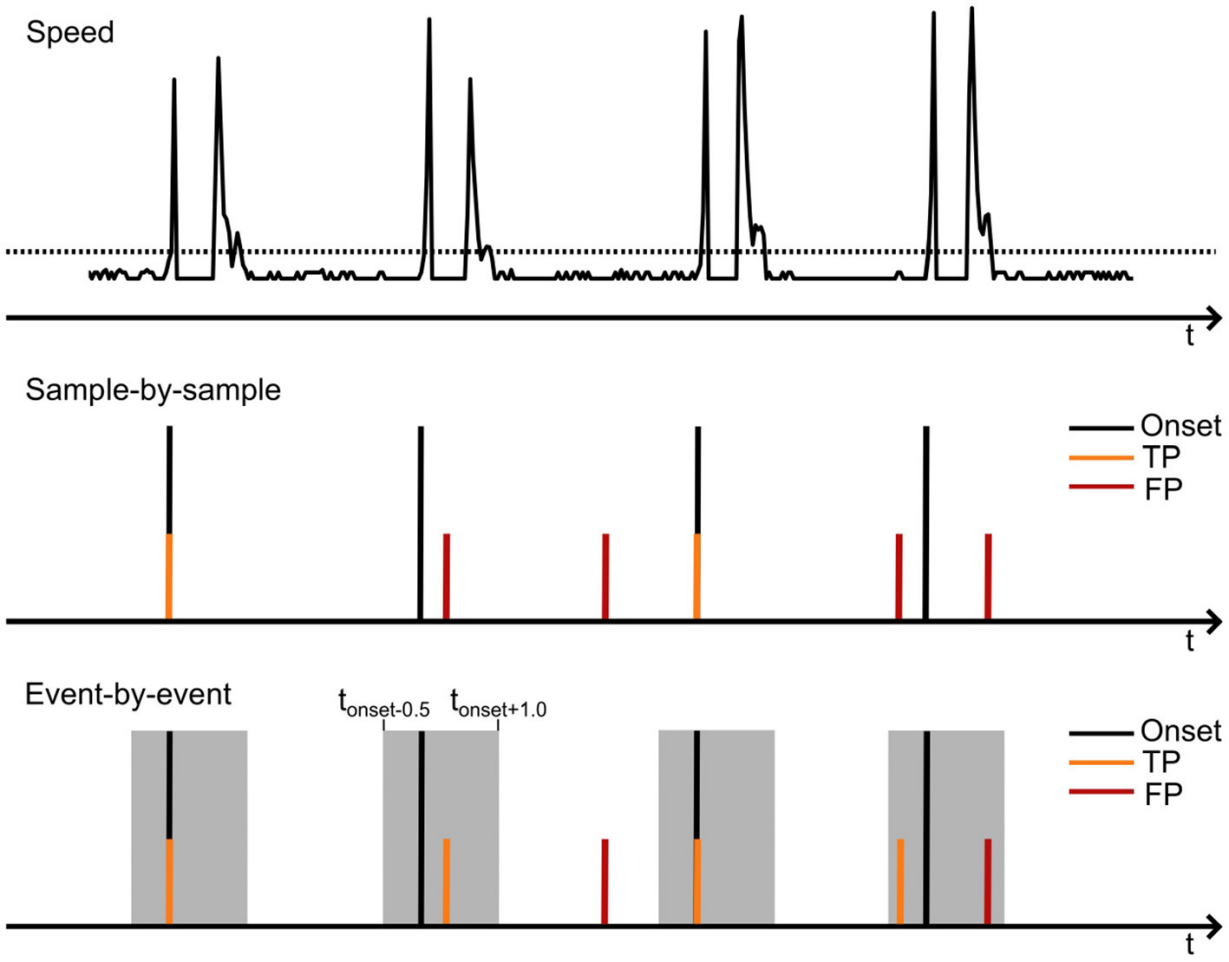
Schematic of the detection of the movement onset from the motion capture data **(top)** and the evaluation of true and false positives on a sample-by sample **(middle)** or event-by-event basis **(lower)**. The upper image illustrates the movement speed extracted from the motion capture data. When the speed exceeds a specified threshold, the movement onset is marked. The second peak, occuring shortly after the movement onset, corresponds to the movement offset.

We first trained the SGEYESUB algorithm (Kobler et al., 2020) on data from the eye-movement paradigm to obtain a matrix which could be used to remove eye movement artifacts. Following this, the EEG data of cue-based and self-paced runs was filtered and re-referenced. We applied a bandpass filter (Butterworth, 4^*th*^ order) between 0.5 and 70 Hz and applied an affine transformation of the obtained eye-paradigm matrix to eliminate eye artifacts. Low-frequency time-series features were extracted by lowpass filtering the data to 3.5 Hz (Butterworth, 4^*th*^ order) and downsampling the data to 10 Hz. Finally, the signals were re-referenced to the common average of all EEG channels. For labeling the training data, we further extracted the actual movement onset from the motion capture data by detecting the time at which the speed of the movement ($s=\sqrt{v_{x}^{2}+v_{y}^{2}}$) exceeded a threshold. An example of the movement onset detection is displayed in Figure 2 (top). Since the movement is shortly paused, one movement consisted of two distinct peaks in the speed. Thus, the movement onset was defined as the first threshold crossing of two consecutive crossings.

### 2.4 Decoder training and evaluation

A binary shrinkage linear discriminant analysis (sLDA) (Blankertz et al., 2011) was trained to identify the initiation of movement based on labeled windows of EEG data. sLDA is a method that is commonly used in the classification of movement-related data from EEG and has been successfully applied to multiple offline and online classification problems (Lotte et al., 2018; Suwandjieff and Müller-Putz, 2024; Ofner et al., 2019; Pereira et al., 2021, 2017). The model was implemented and trained in Matlab (Matlab R2024a, Mathworks Inc. USA) (Dwinnell, 2025). Each window was constructed from the low-pass filtered data *n*_*window*_ samples prior to a time point *t*. Windows were advanced with a stride of one sample. Only windows where *t* corresponded to a time point *t*_*onset*_ + *d*, describing the time of the movement onset *t*_*onset*_ with the addition of a detection delay *d*, were labeled as *movement* while all other windows were labeled as *no movement*. This delay was introduced to include sufficient information from EEG before making a prediction and thus reduce false movement detections. The binary sLDA was trained for multiple values of *n*_*window*_ and *d* on the data of the cue-based runs. To determine optimal values for the number of samples of a window *n*_*window*_ and the detection delay *d*, we applied a 3-fold cross-validation approach in a runwise fashion, where one cue-based run was used for training while the remaining two runs served as the testing set. The shrinkage coefficient of the sLDA was found using the the quadratic-inverse shrinkage estimator method of Ledoit and Wolf which provides a closed-form solution (Ledoit and Wolf, 1996, 2022) (Matlab function *QIS.m* from Ledoit et al., 2025). This closed-form estimator eliminates the need to apply cross-validation for selecting the shrinkage coefficient, saving considerable computation runtime. Additionally, it ensures robustness as it does not introduce variation caused by the data split process during cross-validation for regularization coefficient selection. The optimal values of *n*_*window*_ and *d* were subsequently selected using the Matthews Correlation Coefficient (MCC) (Matthews, 1975; Chicco and Jurman, 2020) as the performance measure on the testing set for each participant individually. We selected the MCC because of the class imbalance in the data, where *no movement* labels were much more frequent than *movement* labels. The MCC is a robust metric for handling imbalanced datasets, as it accounts for true and false positives and negatives, offering a more reliable assessment than accuracy alone and has been successfully employed in our previous work (Kostoglou et al., 2024). The optimal values for *n*_*window*_ and *d* were then selected by maximizing the cross-validated MCC. Finally, an sLDA model was built using these optimal parameters and the complete training data from all three runs.

We finally evaluated the model's accuracy in predicting movement onsets during the self-paced run. Unlike the training phase, where performance (i.e., MCC) was assessed sample-by-sample, the evaluation was conducted on an event-by-event basis (Townsend et al., 2004). While the MCC finds an optimal performance point based on the balance of true and false predictions, it does not account for usability in an online BCI. Thus, the event-by-event-based analysis allowed us to calculate both the true positive rate (TPR) and the rate of false positives per minute (FP/min). The TPR represents the percentage of true movement onsets predicted correctly, while the rate of FP/min measures the number of false positives occurring within a one-minute period. TPR and FP/min values were computed with a strict temporal resolution of [−0.5; + 1]*s* around the actual movement onset. Additionally, a refractory period of 1 s was incorporated to minimize the impact of closely spaced false positives. Figure 2 shows the detection of the movement onset from the speed of the motion as well as the evaluation of true and false positives based on a sample-by-sample or event-by-event basis. The sample-by-sample-based evaluation utilized in the training of the model regarded only those movement predictions as correct which were detected at the exact time of the movement onset. In the event-by-event-based evaluation, detected movements in a window of [−0.5; + 1]*s* around the movement onset were selected as correct detections, if they were not preceded by another detected motion in the same window. To ensure consistent analysis across all participants, the model was finally tuned to optimize performance based on a maximum rate of FP/min. This *calibrated FP/min* rate was achieved by adjusting the probability threshold of the sLDA model's output individually for each participant, ensuring that the rate of FP/min remained below a predefined value while also maximizing the TPR in the self-paced run. The rate was set to 0.5, 1 or 2 FP/min to analyze the impact of different FP/min thresholds on the TPR.

### 2.5 Analysis of neural correlates and behavioral analysis

We investigated the neural correlates of executed movements during cue-based and self-paced runs by analyzing low-frequency time-series features. The neural response elicited during movements in the low-frequency component of the EEG is typically referred to as movement-related cortical potential (MRCP). MRCPs are time- and phase-locked neural correlates of movement that occur in the time-domain EEG. Typically, they exhibit a negative deflection up to 2 s prior to the movement onset and reach their maximum negativity at the time of the movement onset (Shibasaki and Hallett, 2006). Post movement, a positive potential emerges before returning to baseline. MRCPs are generated in central motor areas contralateral to the movement. Apart from executed motions, imagined or attempted movements similarly produce MRCP patterns (Pereira et al., 2017, 2018). To identify whether MRCPs during cue-based runs differed significantly from those during self-paced movement execution, we investigated the trials of all participants ±1.0 s around the movement onset. For every channel and time sample, we identified significant differences by applying a Mann-Whitney U test (Mann and Whitney, 1947) and corrected for multiple comparisons using the Bonferroni method (Dunn, 1961). In contrast to the extracted low-frequency features for decoder training, we did not apply any re-referencing to the signals during the analysis of neural correlates. Further, we examined whether the dissimilarity between MRCPs elicited during cue-based paradigm runs and MRCPs elicited during self-paced movement execution had an impact on the obtained performance. We calculated the average MRCP ±2.0 s around the movement onset of all cue-based runs and of the self-paced run per participant. To estimate a metric of dissimilarity between MRCPs in cue-based and self-paced runs, we obtained the normalized root mean squared error (NRMSE) between the MRCPs per participant. We then calculated the Pearson correlation coefficient between the NRMSE and the TPR of all participants. This analysis was repeated for all channels over central motor areas (FC3, FC1, FCz, FC2, FC4, C3, C1, Cz, C2, C4, CP3, CP1, CPz, CP2, CP4).

Apart from time-series correlates of movement, we also analyzed the response of the cue-based and self-paced movements in the time-frequency domain in the form of event-related desynchronization and synchronization (ERDS) patterns. All types of executed, but also imagined or attempted, movements result in changes in band power relative to a reference period prior to the movement. These changes occur in the form of desynchronization in alpha and beta frequencies shortly before the onset of the motion and during the movement followed by synchronization in beta frequencies after the movement (Pfurtscheller and Silva, 1999). We extracted time-frequency signals for 36 overlapping center frequencies between 5 and 40 Hz with a bandwidth of 2 Hz and calculated ERDS maps as described by Graimann et al. (2002). The reference interval was set to [−2; −0.5] s relative to the movement onset. Significant ERDS patterns were identified using a bootstrap algorithm (Graimann et al., 2002). ERDS maps were averaged across all participants within a ±4 s window around the movement onset in cue-based and self-paced runs.

We further investigated the behavior of participants regarding the accuracy with which they initiated their movement relative to the cue and the difference of the ITI during the self-paced run and the cue-based runs.

## 3 Results

**Figure 3:**
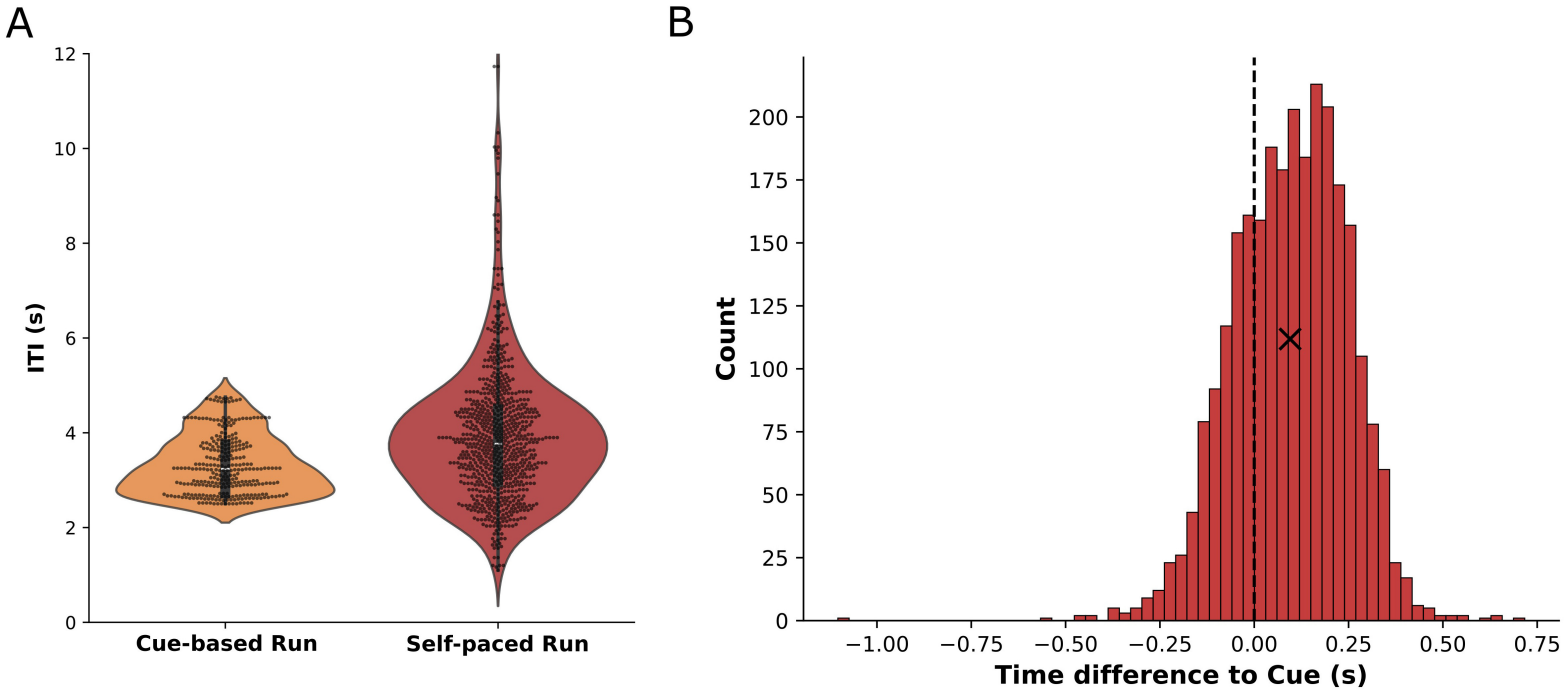
**(A)** Inter-trial-intervals for cue-based runs (orange) and self-paced runs (red). The ITI distribution for cue-based runs is shown based on the cues per run (100 trials) while the ITI distribution for self-paced runs comprises the time between movements from all participants in every self-paced run. **(B)** Histogram of the time difference between the actual movement onset and the cue during cue-based runs. A positive difference indicates that participants executed the movement later than intended by the cue. The mean value (+95 ms) is indicated by a cross.

The behavioral analysis of the movement timing during the self-paced run revealed that participants executed their movements with an average ITI of 3.9 s (standard deviation: ±1.4 s) compared to the average ITI of 3.3 s (standard deviation: ±0.6 s) during the cue-based runs. These results are depicted in Figure 3A where the distribution of ITI per cue-based run is shown (orange) together with the overall distribution of the ITI of all participants during self-paced runs (red). The standard deviation of the mean ITI per participant was 0.8 s while the mean of the standard deviations per participant was 1.1 s, indicating that, while participants utilized a larger spread of ITI during the self-paced run, they had similar average ITI. Figure 3B shows the histogram of differences between the cue and the actual movement onset as detected from the motion capture data. On average, participants executed their movement 95 ms (standard deviation: ±151 ms) after the cue.

**Figure 4:**
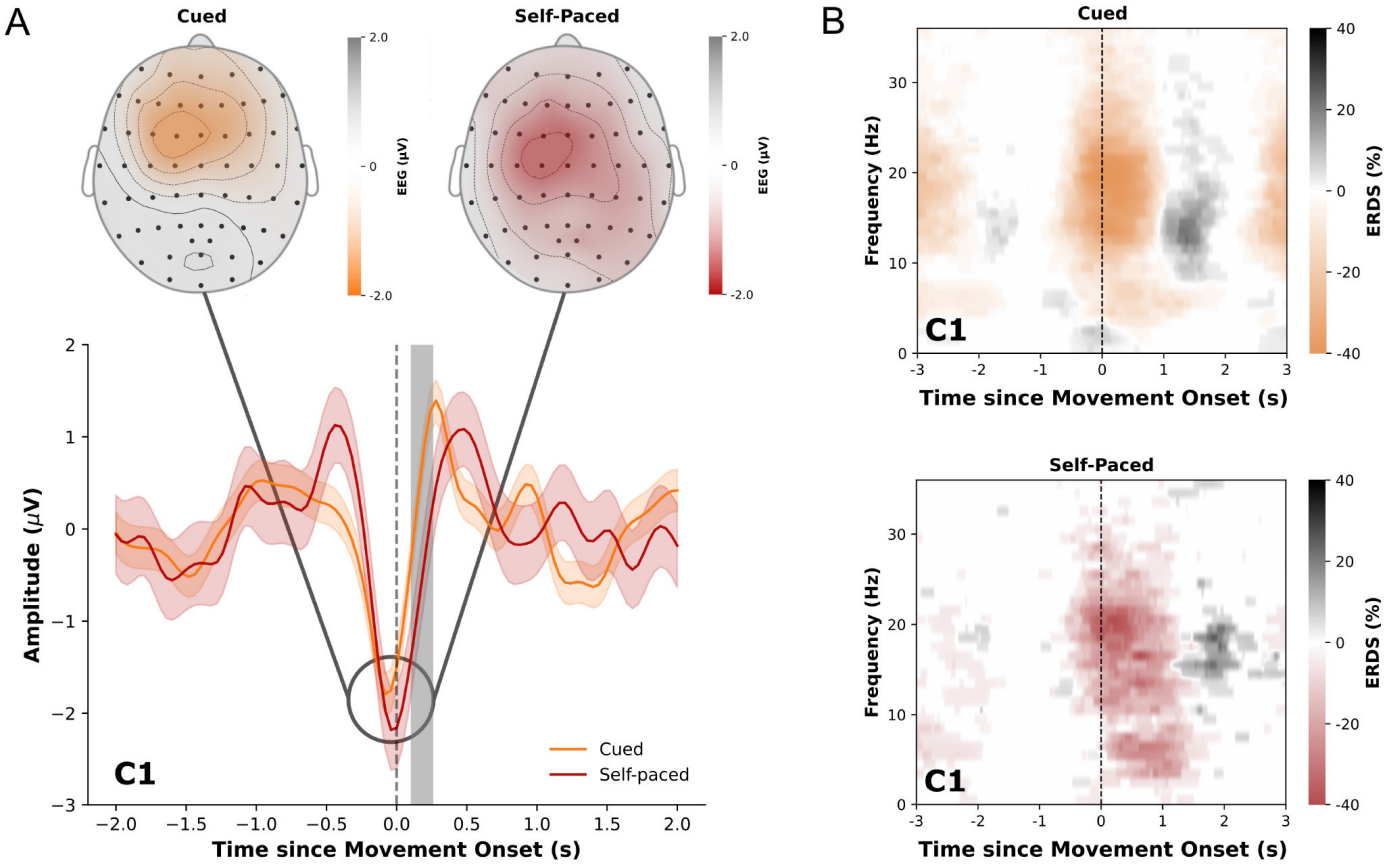
**(A)** Averaged low-frequency time-domain data ±2.0 s relative to the movement onset (gray dashed line) in cue-based (orange) and self-paced (red) runs for channel C1. The data is displayed as the average from all trials and participants together with the standard error. Statistically significant differences (*p* < 0.05) are marked in gray. Upper diagrams display the topological distribution of the amplitude of the averaged low-frequency time-domain data at *t*= -0.1 s relative to the movement onset for all channels. **(B)** ERDS patterns (5-40 Hz) for cued and self-paced movements ±3 s relative to the actual movement onset. Desynchronization is marked in orange/red and synchronization in gray.

The analysis of low-frequency time-domain neural correlates of cue-based and self-paced movements revealed significant differences between the two conditions in multiple channels. Centro-parietal channels showed significant differences ±500 ms around the movement onset while channels over the central motor area exhibited significant differences mostly around 200 ms after the movement onset. Grand-average MRCPs of all participants for the two conditions in channel C1 are shown in Figure 4A together with statistically significant differences highlighted in gray. The figure also displays the topographical distribution of MRCPs for both cue-based and self-paced runs 100 ms prior to the detected movement onset. Detailed figures displaying the distribution of amplitude and differences between the conditions with highlighted statistical significances for multiple timepoints can be found in Supplementary Figures S1, S2. For individual paricipants, the MRCPs can be found in Supplementary Figure S3.

The obtained ERDS maps for cue-based and self-paced movements show typical desynchronization of alpha and beta frequencies around the movement onset and synchronization of beta frequencies after the end of the movement. These patterns are shown in Figure 4B for channel C1 in cue-based and self-paced runs. ERDS maps for all channels can be found in Supplementary Figure S4 for cue-based runs and in Supplementary Figure S5 for self-paced runs.

**Figure 5:**
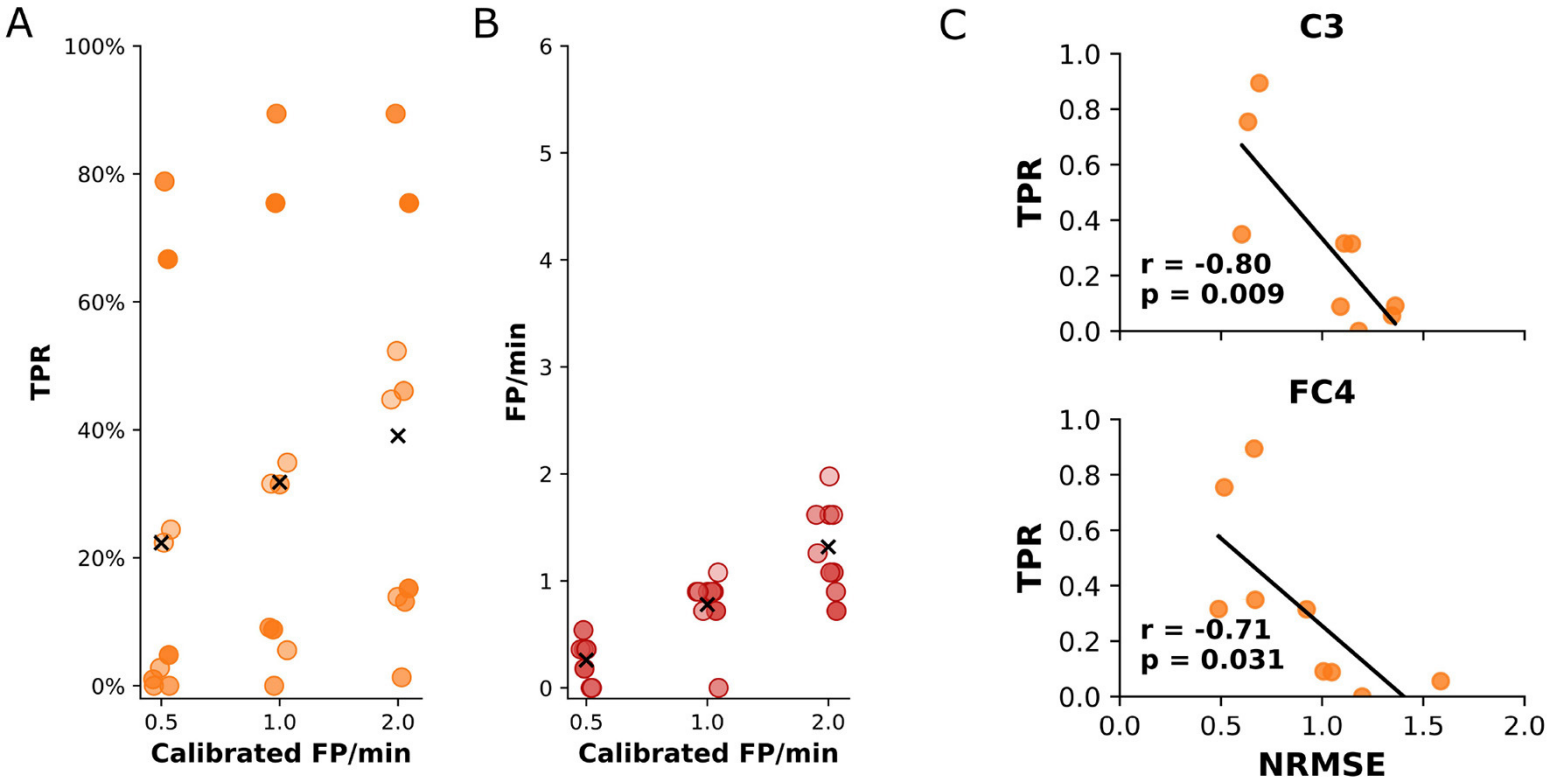
**(A)** True positive rate of the detected movements during the self-paced run for every participant. The results are shown for three different calibrated rates of false positives per minute. **(B)** The obtained rates of false positives per minute of the detected movements during the self-paced run for every participant. The results show the actual number of false positives per minute that occurred during the self-paced run for three different calibrated maximum false positives per minute. **(C)** Statistically significant (*p* < 0.05) correlations between the TPR during the self-paced run and the NRMSE. The correlation was estimated using the Pearson correlation coefficient. Correlation coefficients (*r*) and *p*-values (*p*) are displayed.

As outlined earlier, the optimal values *n*_*window*_ and *d* were determined by maximizing the cross-validated MCC on the training runs. The final performance of the decoder model was evaluated on the self-paced run for each participant individually on an event-by-event basis, using the TPR and the rate of FP/min as metrics. We individually adjusted the probability threshold of the sLDA model's output for each participant, aiming to keep the rate of FP/min below a fixed value while maximizing the TPR during the self-paced run. The average TPR of the model for a maximum rate of 1 FP/min was 31.8% (standard deviation: ±29.8%) with a final occurrence of 0.8 (standard deviation: ±0.3) FP/min during the self-paced run. For a calibrated rate of 0.5 and 2.0 FP/min, the average TPR was 22.3% (standard deviation: 28.5%) and 39.1% (standard deviation: 28.7%), respectively. The actual occurrence of FP/min was 0.3 (standard deviation: 0.2) and 1.3 (standard deviation: 0.4). The results for all participants are summarized in Figures 5A, B. The dependence of the TPR and actual rate of FP/min on the sLDA output probability threshold for all participants is shown in Supplementary Figure S6.

The analysis of the relationship between the TPR and the dissimilarity between MRCPs elicited during cue-based and self-paced runs (NRMSE) per participant revealed a significant negative correlation for channels C3 (*r* = −0.80, *p* = 0.009) and FC4 (*r* = −0.71, *p* = 0.031). In practical terms, this means that when the MRCP patterns recorded at C3 and FC4 during self-paced movements closely matched the patterns observed during training, the decoder was better at correctly detecting movements. Conversely, when there was greater dissimilarity, the decoder's detection performance declined. The results are shown in Figure 5C. For all other channels, the correlation was similarly negative (*r* < 0.0) but not significant (*p*>0.05). The full results of the correlation for all channels can be found in Supplementary Figure S7.

## 4 Discussion

In this work, we presented a novel experimental paradigm aimed at reducing the time required for the collection of training data for movement-based BCIs while also addressing challenges associated with traditional cue-based methods (Ofner et al., 2019). The close resemblance of neural correlates of movement during cue-based runs with those from self-paced movements together with the fast pace at which cue-based movement can be executed is novel and enables the collection of suitable training data for asynchronous movement-based BCIs in very short time. The paradigm utilizes a rotational, cross-based cue (Suwandjieff and Müller-Putz, 2024) where a fixed cross (fixation cross) and a rotating cross (rotation cross) are displayed on a screen. Participants are instructed to execute a specific movement when both crosses overlap, serving as the cue for movement initiation. While Suwandjieff and Müller-Putz (2024) introduced the general concept of the overlapping, rotating cross, they utilized the paradigm solely for synchronous classification. In this traditional synchronous paradigm, participants experienced long waiting times between movements, and the continuous data was interrupted by visually evoked potentials caused by the appearance and disappearance of the crosses between trials. In our current work, we adapted the paradigm to feature a continuously rotating cross, eliminating the disappearance of the crosses between movements. This adjustment removed the influence of abrupt visual appearances and enabled the utilization of uninterrupted, continuous data from multiple, consecutive movement executions. Further, we introduced variable inter-trial-intervals by continuously adapting the velocity of the rotating cross.

Thus, our proposed setup allows for continuous data collection with minimal breaks, thereby reducing the overall time needed for the collection of training data. In traditional cue-based paradigms, data collection is not continuous, necessitating breaks between cue presentation and movement execution to minimize visual effects. This leads to long inter-trial times such as described by Ofner et al. (2019) with a minimum of 8.5 s. In comparison, the inter-trial time in the proposed paradigm is on average 3.3 s. For a run of 300 trials, the traditional cue-based paradigms would therefore require a duration of at least 42 min while our proposed paradigm has a duration of about 18 min, thereby reducing the required training time to less than half of the time of traditional paradigms. As a result, the data acquisition process is significantly faster compared to traditional paradigms, making it more suitable for practical applications. This is particularly relevant for individuals with motor disabilities, since they often cannot endure prolonged experimental sessions to collect sufficient training data. By incorporating cue-based movements into a continuous, uninterrupted session, participants also remain in an active state of readiness even during rest periods. This design simulates real-world scenarios in which a BCI user may be in a waiting state, such as preparing to click on a screen or type in a speller in a self-paced and asynchronous manner. This active rest state ensures that the collected data better represents typical BCI usage, where users often shift between periods of active waiting and action.

While the current study relies on the movement onset as detected from motion capture data, the analysis of time differences between the cue and the actual execution of the movement shows that participants generally executed the motions shortly after the cue onset with a low standard deviation. This is relevant especially for the inclusion of motor-impaired participants in the future, since the actual movement onset cannot be inferred in their case. The behavioral results suggest that the training of movement-based BCIs based on data labeled with cue timings is possible.

A key advantage of the proposed paradigm is its ability to generate brain activity that closely resembles the neural patterns observed during self-paced movements, including both MRCPs (Figure 4) and ERDS patterns (Supplementary Figures S4, S5). While there exist significant differences after the movement onset, no such differences were found prior to the movement. Specifically, we did not find any evidence for visual evoked potentials introduced by the proposed paradigm. Further, the obtained ERDS patterns did not suggest any apparent difference between cue-based and self-paced execution. This is a significant improvement over traditional cue-based paradigms, which rely on visual or auditory cues to synchronize movement (execution, attempt or imagination) timing but inadvertently introduce evoked potentials that overlap with movement-related brain signals (see e.g. Ofner et al., 2019). Interestingly, Suwandjieff and Müller-Putz (2024) found significant differences also prior to the movement in their implementation of the rotational cue. This suggests that, by incorporating a continuous rotation, visual influences of the cue could be further reduced. By minimizing these artifacts, the current approach captures more natural movement-related brain signals, which are essential for training accurate BCI decoders. However, even after minimizing the effects of the cues, our analysis revealed differences between MRCPs elicited during self-paced and cue-based runs. This suggests that, despite reducing cue-related artifacts, distinct brain dynamics between voluntary (self-paced) and externally driven movements persist. These residual differences could still impact the accuracy of BCI systems, potentially limiting the ability to consistently decode motor intentions across different conditions. Pereira et al. (2018) also investigated the neural dynamics associated with different types of movement imaginations. They also found that externally-cued movement imaginations elicited distinct EEG patterns compared to self-paced movement imaginations. Specifically, externally-cued movements were associated with increased activation in the parietal and occipital regions, reflecting the processing of external stimuli. This underscores the need for further refinement in BCI designs to address these neural variations, ensuring more reliable performance.

To evaluate the feasibility of the proposed paradigm and approach to collect training data for movement-based BCIs, a decoder was trained on the collected data and the performance was tested on self-paced data. The model achieved an average TPR of 31.8% for detecting movement at a calibrated rate of 1.0 FP/min, though performance varied greatly across participants. In practical terms, for almost every three attempted movements, approximately one was successfully identified by the system as an intentional movement. The remaining 2 out of 3 were missed, resulting in false negatives. The low rate of 1.0 FP/min indicates that these missed detections did not result in incorrect detections but were simply not detected. While some participants demonstrated high TPR, others showed much lower values, underscoring the impact of individual variability. Since higher similarity between MRCPs in self-paced and cue-based runs correlated with higher performances in the self-paced task, we suggest that these individual performance disparities could be due to differences in neural responses of participants to the utilized cue. Thus, it needs to be investigated whether the neural response during movements of participants is closer to the response during self-paced movement execution for other cues which could therefore be more useful.

While other studies on the asynchronous detection of movements show promising results, a comparison of the achieved performance to these studies is difficult, since the calibrated rate of FP/min has a large impact on the obtained TPR, as has been shown above. Zhang et al. (2024) investigated the accuracy of predicting movement onset using both synchronous and asynchronous classification methods, specifically focusing on MRCPs generated from hand movements at varying speeds. For synchronous classification, where external cues were used to time movement predictions, the average accuracy across six healthy participants was 68.7% for fast movements and 70.6% for slower movements. Asynchronous detection, however, was tested only in one of the six participants, utilizing 320 trials for training over a duration of approximately two hours, focusing on real-time predictions without external cues. Niazi et al. (2011) reported an average TPR of 55.4% and 11.4 FP/min among 15 healthy subjects during asynchronous ankle dorsiflexion, utilizing 30 training trials collected over approximately 10 minutes. However, they demonstrated that spatial filtering techniques not only improved detection performance, but also reduced the number of FPs. Xu et al. (2014) used a manifold learning technique combined with LDA which resulted in TPRs exceeding 80% and less than 2 FP/min in nine healthy participants based on 30 training trials conducted over approximately 10 min. Hasan and Gan (2010) employed both supervised and unsupervised approaches to detect wrist extensions in five healthy individuals and found that unsupervised techniques led to increased asynchronous detection performance. Fatourechi et al. (2007, 2008) proposed extracting both temporal and spectral EEG features based on three neurological phenomena: MRCPs, changes in the power of mu rhythms and changes in the power of beta rhythms. For asynchronous finger flexion detection they reported low FP rates at a modest TPR for four subjects, showing on average 10 FP/min at a TPR of 41.1%, respectively, for the initial BCI system design (utilizing approximately 320 training trials recorded over a period of almost 50 min), and 0.7 FP/min and a TPR of 56.2% for the improved version. The results from Fatourechi et al. (2007, 2008) align with those presented in the current study. While the rates of FP/min are similarly low, the current study did not achieve an equivalently high TPR. However, herein, the primary focus was on the experimental paradigm and data collection, rather than on selecting features or decoders for optimal TPRs. While decoding plays an important role, our goal in this work was to establish a robust and efficient paradigm for data collection that better captures natural brain dynamics.

When selecting different features, one limitation to consider is that spectral features may require a longer inter-trial interval than those used in the current study to accurately capture their dynamics since ERDS patterns are observed on a larger and more delayed time scale compared to MRCPs during neural processing. However, this aspect requires further investigation from both experimental and analytical perspectives. Aside from different features, a limitation of the current study is the lack of investigation of different models to increase the classification performance. Future work will test multiple state-of-the-art models to achieve an increase in the TPR while obtaining a similarly low rate of FP/min. The main limitation of the current study is the lack of evaluation within an online paradigm. While we showed that the collected data is feasible for training a model to detect movements during self-paced motion execution, it is currently not clear whether this also applies to online paradigms such as spellers. Our next step is therefore to apply this framework to an online detection task within a spelling application, providing the opportunity for real-time feedback and user engagement. This approach will enable us to assess the effectiveness of our MRCP-based decoder for spelling, allowing users to make selections on highlighted columns or rows in a concept similar to P300 spelling (Farwell and Donchin, 1988). Moving forward, we aim to improve our decoding system by using cue onsets for labeling the data rather than relying on movement onsets. For individuals who cannot perform physical movements, the cue onset is the only reliable marker for detecting motor intention. This adjustment is thus crucial for developing BCIs that support motor-impaired individuals.

While the paradigm was designed and tested using non-invasive EEG, its application is similarly important for invasively recorded neural signals. By accelerating and improving the collection of training data, the proposed paradigm has implications also for real-world applications and patients which use BCIs for daily communication. It is therefore relevant to examine the behavior of the proposed paradigm during invasive recordings of neural data and investigate the usage in invasive BCIs.

## Data Availability

The raw data supporting the conclusions of this article will be made available by the authors, without undue reservation.
