# Supplementary material for: A novel paradigm for fast training data generation in asynchronous movement-based BCIs
Source: Front Hum Neurosci. 2025 Feb 11;19:1540155. doi: 10.3389/fnhum.2025.1540155 (PMC11850329; doi:10.3389/fnhum.2025.1540155)
Supplement: Supplementary file 1 [file Data_Sheet_1.pdf]

Supplementary Material

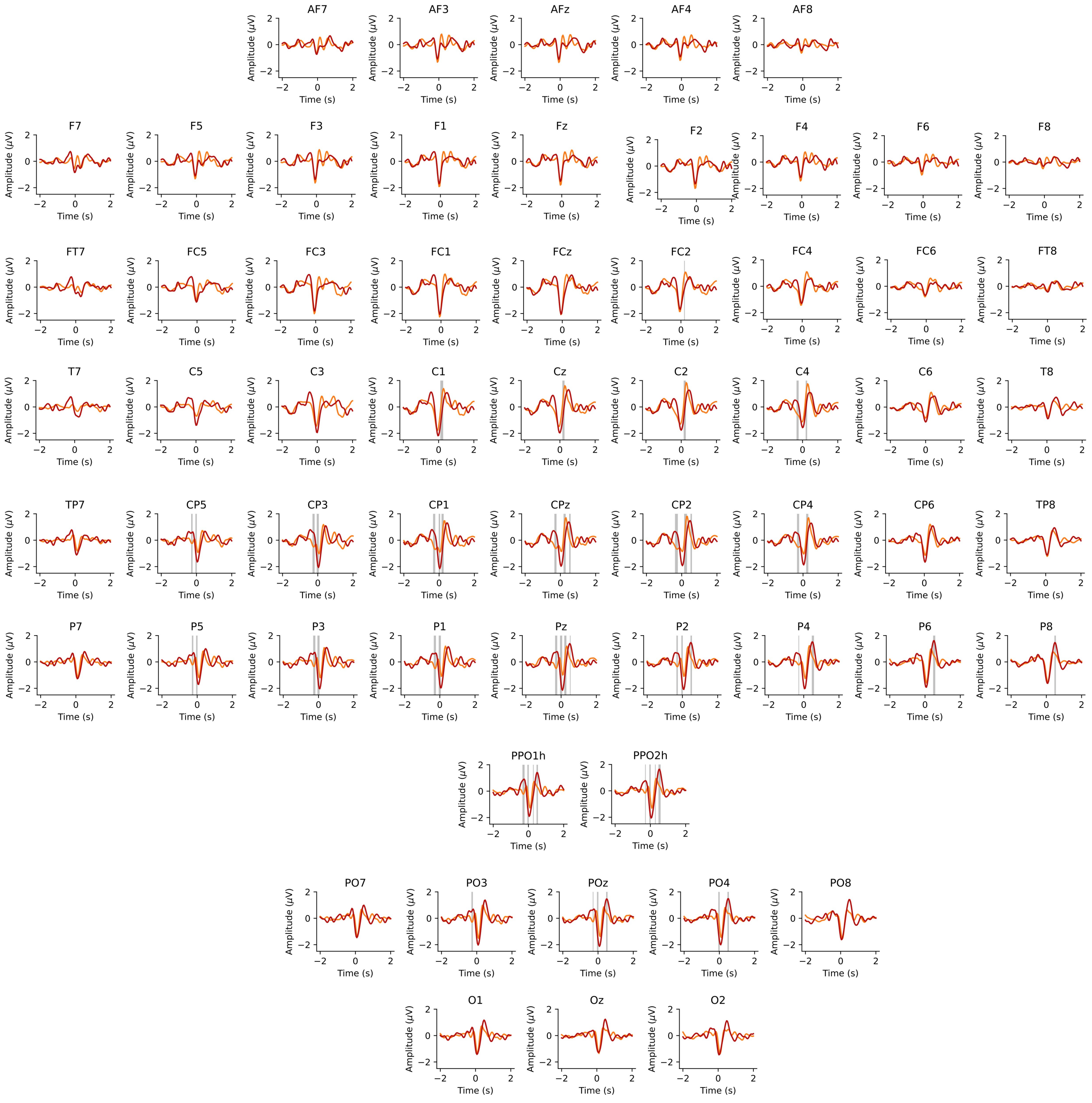

Figure S1. Grand-average MRCPs from all participants and all channels. Statistically significant ($p < 0.05$) differences between the neural response of movement execution during cue-based and self-paced runs are marked in grey. Images are ordered according to the position of the corresponding channel on the head (10-10 system).

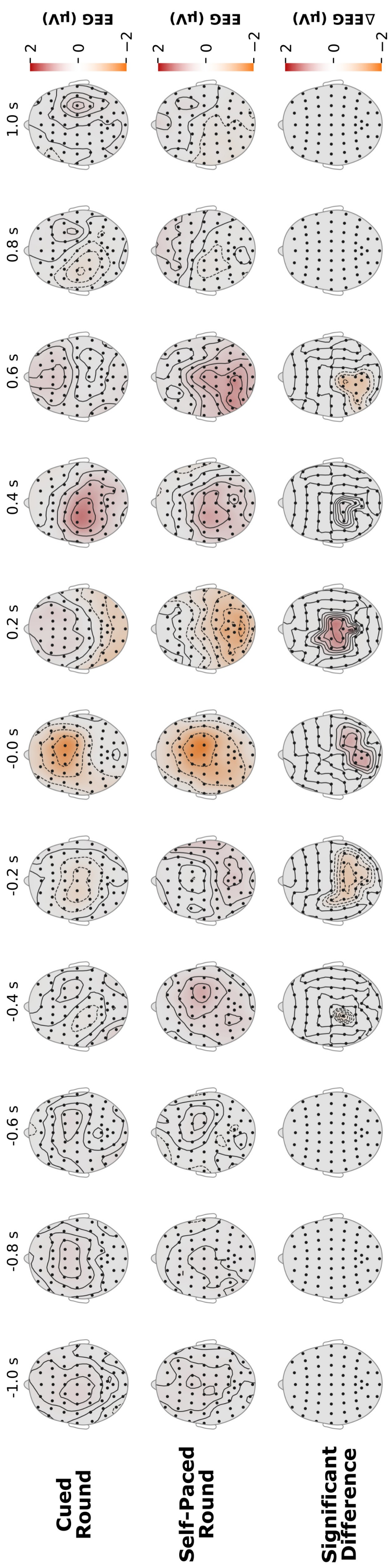

Figure S2. Topographical distribution of the grand-average MRCPs elicited during cue-based (first row) and self-paced (second row) runs as well as the difference between both conditions (third row). For differences between the two conditions, only statistically significant ($p < 0.05$) differences are displayed.

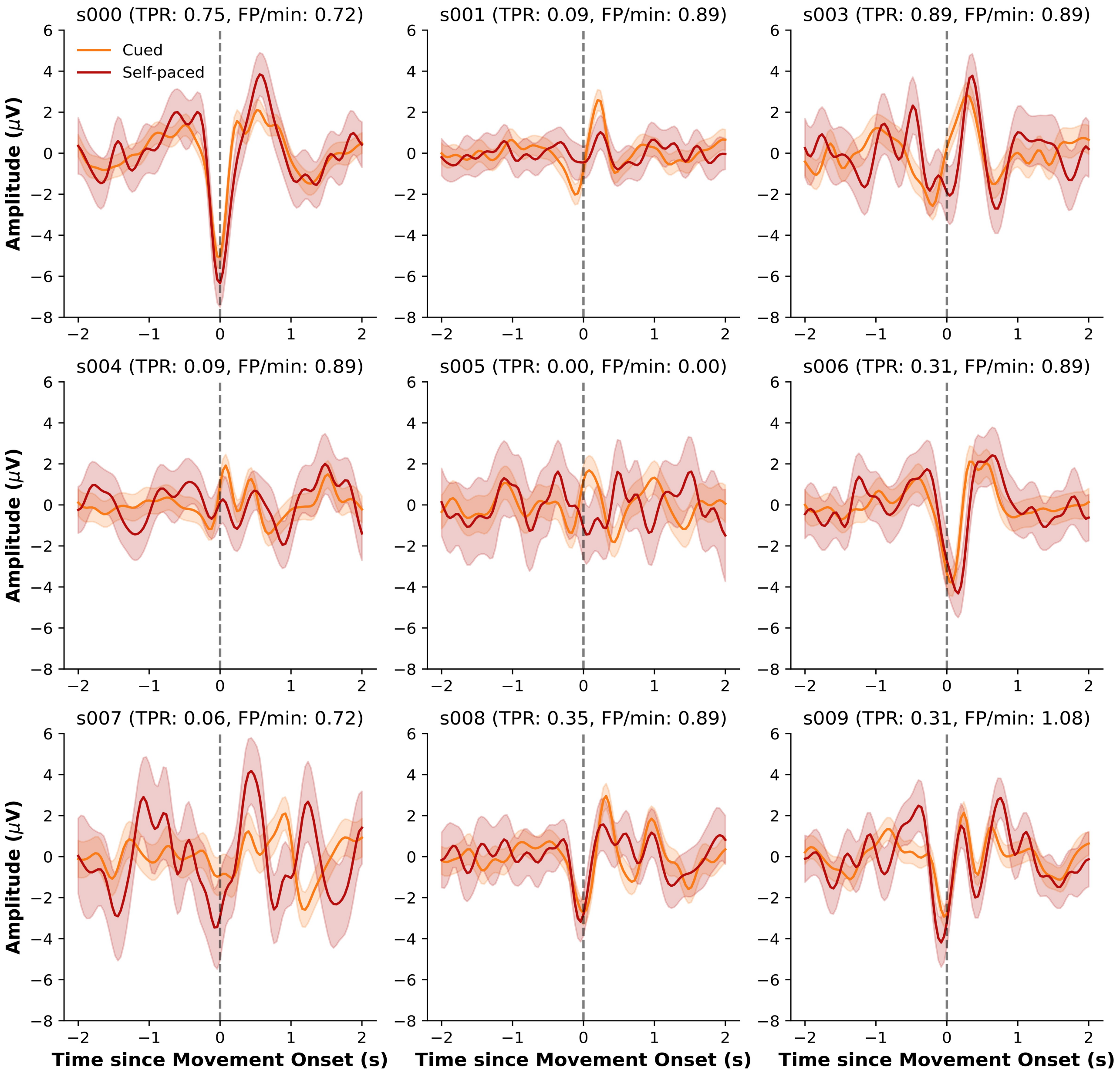

Figure S3. Grand-average MRCPs for each participant $\pm 2.0\,\mathrm{s}$ relative to the movement onset (grey dashed line) in cued (orange) and self-paced (red) runs for channel C1. The standard error is displayed around the averaged data.

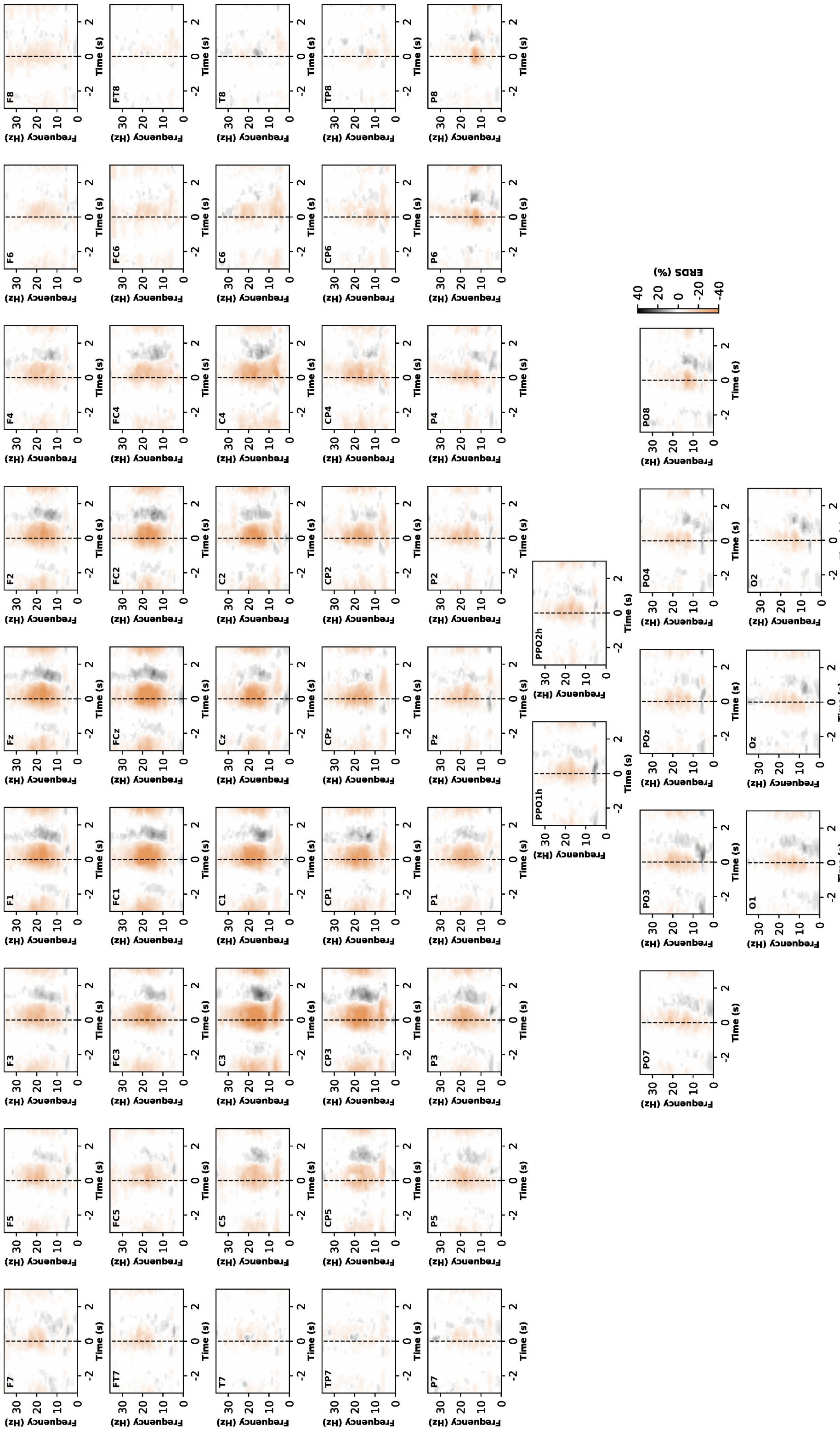

Figure S4. Grand-average ERDS maps for cue-based runs and all except for frontal channels. The time ranges from 3 s prior to the movement onset to 3 s after the movement onset.

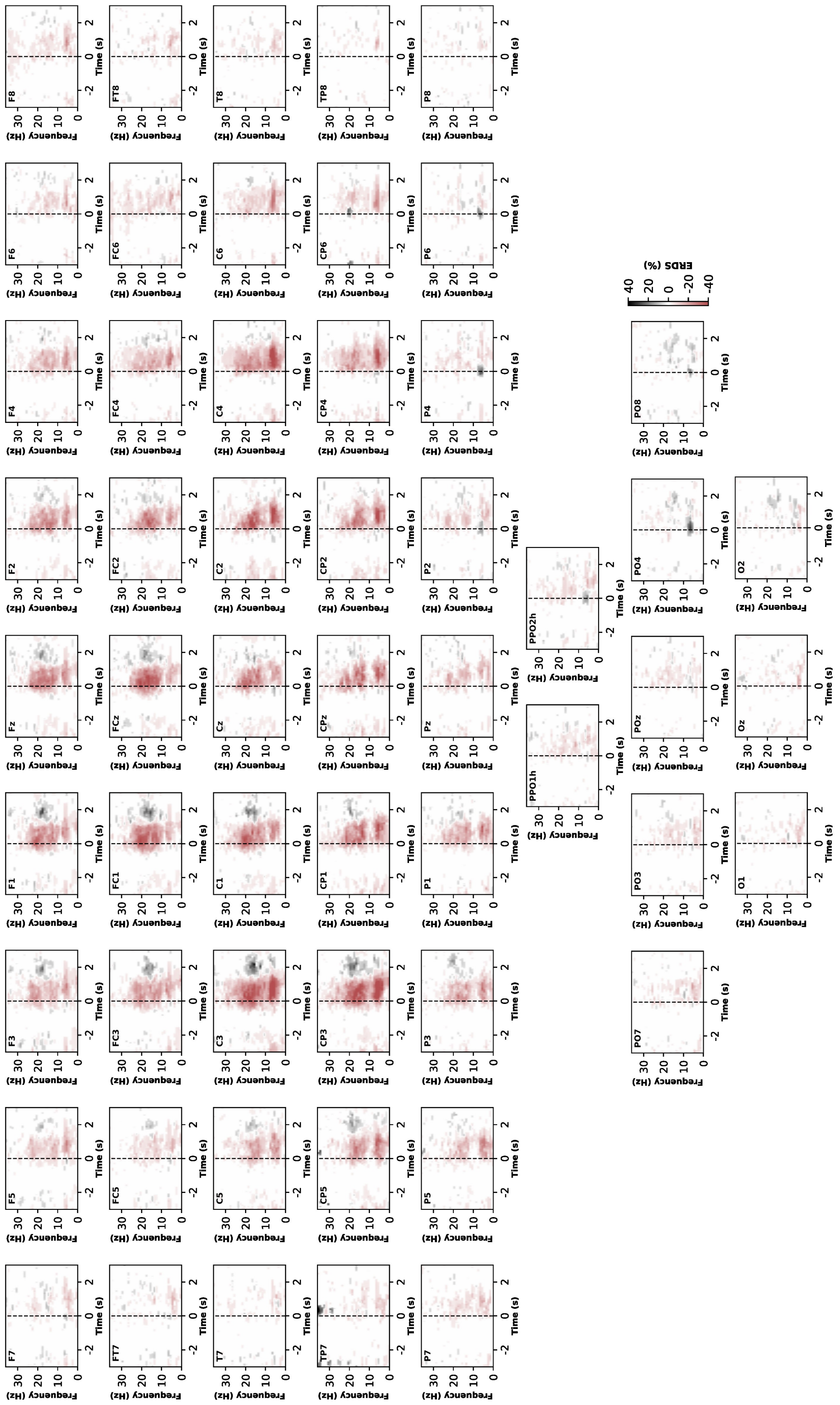

Figure S5. Grand-average ERDS maps for self-paced runs and all except for frontal channels. The time ranges from 3 s prior to the movement onset to 3 s after the movement onset.

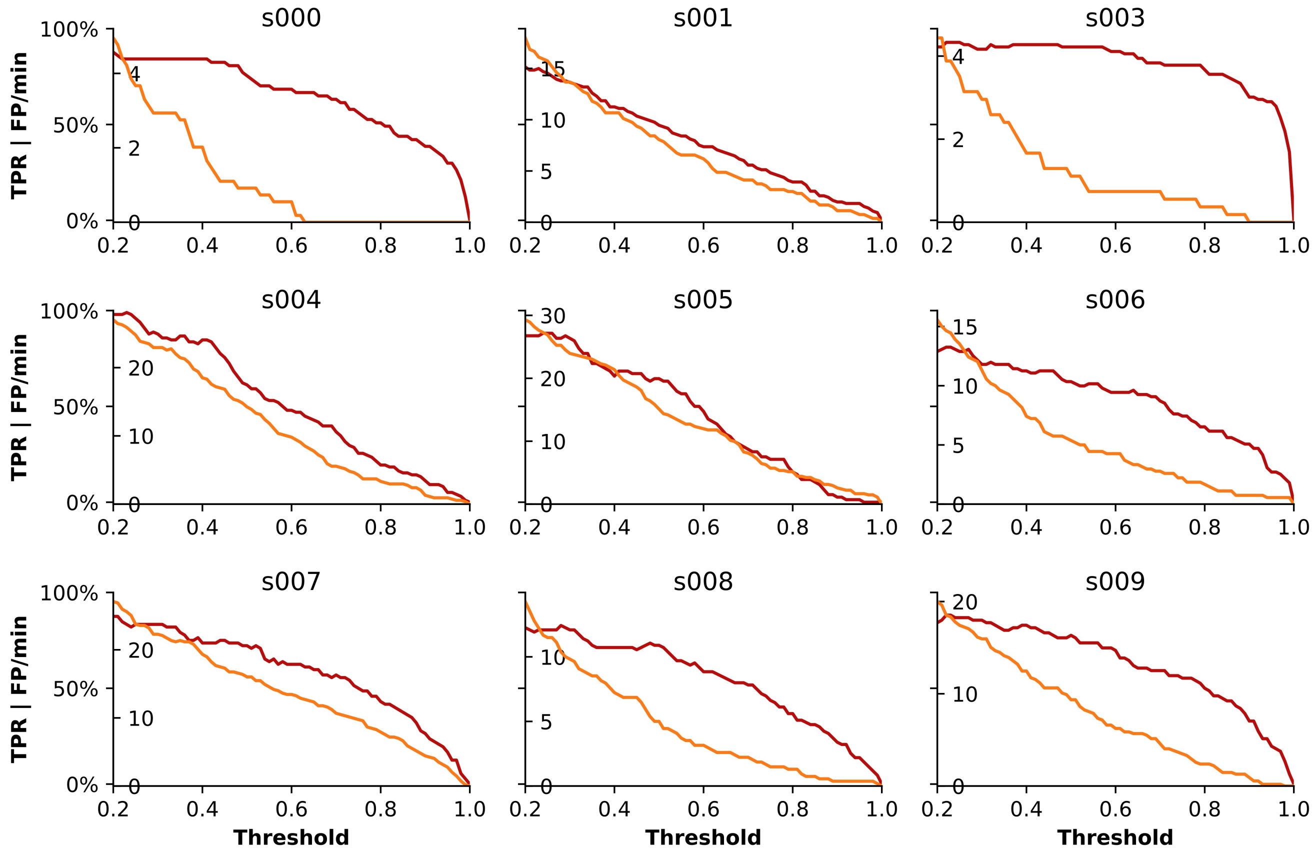

Figure S6. Dependence of the TPR and FP/min on the probability threshold p of the detection model. The performance was evaluated for $0.2 \leq p \leq 1.0$ in steps of 0.01. For every participant, the TPR is depicted in red and the corresponding rate of FP/min in orange.

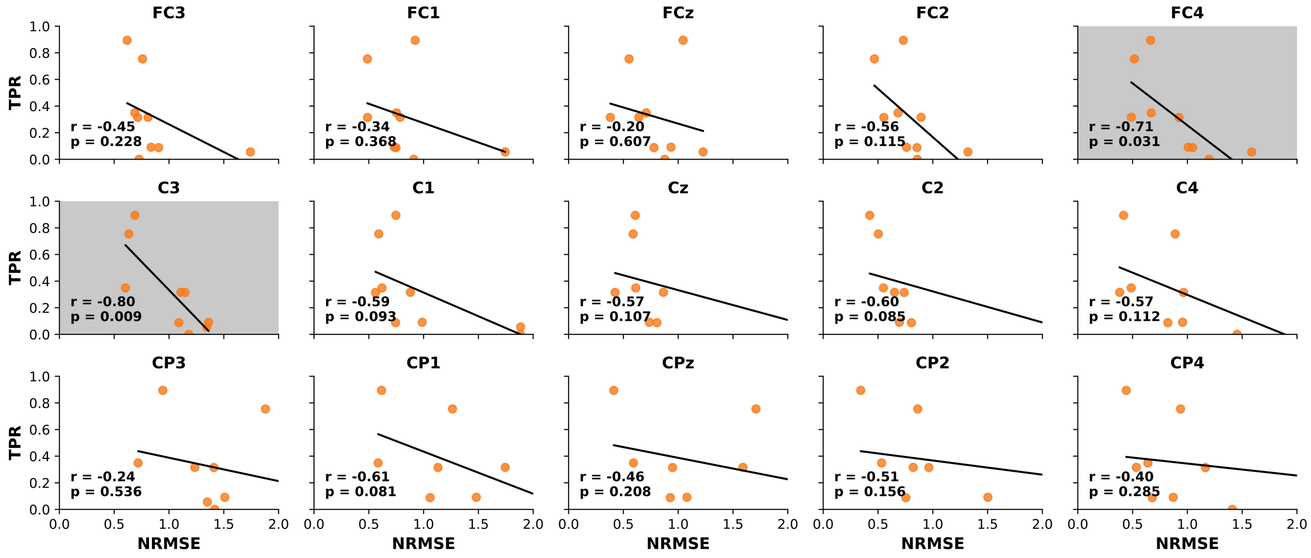

Figure S7. Correlation between the TPR during the self-paced run and the NRMSE for all channels over central motor areas. The correlation was estimated using the Pearson correlation coefficient and corrected for multiple comparison using the Bonferroni method. Correlation coefficients (r) and p-values (p) are displayed.
